# P12 A case of MDA5-positive Juvenile Dermatomyositis complicated by Macrophage Activation Syndrome

**DOI:** 10.1093/rap/rkac067.012

**Published:** 2022-09-28

**Authors:** Elizabeth Twynam-Perkins, Neil Martin

**Affiliations:** Royal Hospital for Children, Glasgow, United Kingdom; Royal Hospital for Children, Glasgow, United Kingdom

## Abstract

**Introduction/Background:**

Here we describe a case of juvenile dermatomyositis (JDM) complicated by macrophage activation syndrome (MAS), and the approach taken to treatment. Treatment with pulsed IV methylprednisolone was inadequate to control hyperinflammation in this case, so other treatment strategies were introduced. Macrophage activation syndrome appears to be less common in JDM than in systemic JIA or SLE, but it may be more frequent in MDA5-positive JDM.

**Description/Method:**

An 11-year-old boy presented to paediatrics with a 6-week history of rash, mouth ulcers, anorexia and myalgia. He was previously well, with mild eczema only.

On examination, he had rash affecting his face, periocular area, elbows and hands, with Gottron’s papules, plus arthritis of ankle and PIP joints. He had mild proximal muscle weakness with CMAS 45/52. JDM was diagnosed based on typical rash, proximal muscle weakness, raised muscle enzymes, muscle MRI and positive anti-MDA5.

Echocardiography showed normal cardiac function whilst PFTs showed no evidence of interstitial lung disease.

Blood results are shown in table 1. Findings included raised muscle enzymes, transaminitis, leucopenia, mild thrombocytopaenia and hyperferritinaemia. Two weeks previously, his FBC was normal.

IV methylprednisolone was started on day 2 of admission, however he remained persistently pyrexial, developing a rising ferritin and worsening transaminitis. This pattern was recognised as MAS.

IV immunoglobulin was started on day 7, in addition to methotrexate and hydroxychloroquine. This resulted in resolution of fever and improved bloods. He was discharged on day 10 to continue outpatient management.

After discharge, however, the ferritin climbed again. He was given further methylprednisolone and switched from methotrexate to ciclosporin on day 14. Following this, the features of MAS resolved, with ferritin falling. CMAS normalised within 2 months and his rash gradually improved.

Once ferritin and FBC had normalised, ciclosporin was switched back to methotrexate. He continues on a treatment regimen of hydroxychloroquine, IVIg, methotrexate and reducing oral prednisolone with a plan to reassess after 6 months.

**Discussion/Results:**

MAS, or secondary haemophagocytic lymphohistiocytosis (HLH), is a well-recognised phenomenon in systemic-JIA but may occur in other rheumatological conditions.

The incidence of MAS in JDM is unknown. A systematic review found 12 case reports of MAS in JDM but was unable to estimate incidence or mortality. As with our patient, this case series suggested that JDM-related MAS may be difficult to treat, with methylprednisolone being inadequate in most. Likewise, it found that MAS tended to occur at presentation of JDM. A case series of adults with dermatomyositis complicated by MAS had similar findings.

Cases of MDA5-positive dermatomyositis complicated by MAS have been reported in children and adults, suggesting an association. MDA5 positivity has also been associated with increased risk of interstitial lung disease, potentially suggesting a more severe phenotype.

**Key learning points/Conclusion:**

MAS can occur occasionally in JDM and is most likely to present at diagnosis. In particular it should be considered in a patient presenting with features of JDM and associated cytopaenias or persistent fevers.

Anti-MDA5 positive JDM may be associated with a higher incidence of MAS.

IV methylprednisolone alone may be inadequate to treat MAS in JDM. A combination of IV methylprednisolone, IV immunoglobulin and oral ciclosporin successfully controlled clinical and biochemical features of MAS in this patient with JDM.

**Table 1:** P12 Laboratory results suggestive of MAS

	Day 0	Day 1	Day 4	Day 6	Day 10	Day 14	Day 22
CK	1014	900		169		240	228
Ferritin	1880	2671	1769	5090	1784	3000	989
AST	490	574	321	696	208	273	66
Lymphocytes	1.1	0.9	1.3	0.5	1.0	0.8	1.5

